# Mechanism and Influencing Factors of Low-Carbon Coal Power Transition under China’s Carbon Trading Scheme: An Evolutionary Game Analysis

**DOI:** 10.3390/ijerph20010463

**Published:** 2022-12-27

**Authors:** Feng Liu, Yihang Wei, Yu Du, Tao Lv

**Affiliations:** 1 School of Economics and Management, China University of Mining and Technology, Xuzhou 221116, China; 2 School of Business, East China University of Science and Technology, Shanghai 200237, China

**Keywords:** carbon capture utilization and storage, coal power, carbon trading scheme, electricity marketization

## Abstract

To avoid the energy supply risk caused by the large-scale integration of renewable power with the grid, coal power plants with carbon capture utilization and storage (CCUS) have the potential to play an important role in the transition to a low-carbon electricity system. Based on evolutionary game theory, this study analyzed the equilibrium states, evolutionary trajectory and the corresponding critical conditions between the government and the coal power enterprises in this process. Subsequently, a numerical analysis was conducted. The results showed that the carbon trading scheme can directly promote the upgrade of coal power and this effect can be enhanced by establishing the market-oriented trading mechanism of electricity. The slack quota policy at the current stage can contribute to the emergence of the forerunners adopting the CCUS. The technical level of the CCUS had the most significant influence on the equilibrium of the game system. As technology advances, the game system can rapidly achieve the ideal stable strategy (of non-intervention, low-carbon upgrade). On this basis, the government should promote the synergetic development of the carbon market and power market. Additionally, more financial subsidies should be shifted to R&D (research and development) investment.

## 1. Introduction

In the context of the global trend of combatting climate change, China proposed its own climate strategy in the General Assembly of United Nations in 2020: that China would reach the goals of carbon peak and carbon neutrality by 2030 and 2060, respectively. The introduction of these goals urged the domestic carbon-intensive industries to adjust their current development pattern towards a low-carbon and sustainable one. The power section is the largest producer of carbon dioxide in China and accounts for more than half of the national total emissions [1]. To realize China’s ambitious goals of carbon reduction, the drastic structural change of the electricity mix is essential, and the replacement of coal power by renewable power is regarded as the most promising implementation path [2,3]. Over the past 20 years, China has become the world’s largest producer and consumer of renewable energy [4]. In contrast, based on the deep supply-side reform, the proportion of coal-fired power in China’s installation capacity of electricity decreased from 73% in 2010 to 49% in 2020. Considering the drawback of fluctuant and intermittent output, the large-scale substitution of the coal-fired power by renewable power has significantly increased the instability of the power grid and simultaneously poses a threat to the national power supply security [5,6,7]. The emergence of “electricity shortage” in late 2020 was a classic reflection of such systematic risk.

To avoid the recurrence of such a situation, the Fourteenth Five-Year-Plan of China for a Modern Energy System issued in 2022 pointed out that ensuring a safe and stable power supply is the primary task for the domestic power system [8]. The plan specified the important role of clean and efficient coal-fired power at the current stage, which achieved the improvement of the ideas from low-carbon transition to low-carbon and secure transition. Similarly, against the background of the Russian-Ukrainian War, many European countries also announced the return of coal power to maintain the stability of the energy supply [9,10]. On this basis, the development of carbon capture, utilization and storage (CCUS) technology, which can significantly reduce carbon emissions from coal-fired power while maintaining its advantage in the stability of power supply, makes the sustainable development of coal-fired power possible [11,12]. Therefore, under the dual background of carbon neutrality and the increasing concern for power supply security, the low-carbon transition of coal-fired power is an important part for the remodeling of China’s power system.

By means of carbon trading and reform of the electricity marketization, China is now making full efforts to promote the low-carbon transition of the domestic power system. At present, the emission reduction mechanism driven by carbon costs and mediated by the market-oriented electricity trading scheme is preliminarily established [13]. On this basis, coal power plants are motivated to conduct the low-carbon upgrade to avoid the carbon costs and obtain more generation opportunities, and the CCUS technology is the most representative upgrade solution. Considering that the national carbon trading market has been operating less than two years, its carbon reduction effect still needs to be verified. Additionally, the pricing mechanism of the electricity market has not been completed and so cannot yet fully conduct its filtering function for the low-carbon electricity [14,15]. To make up for the temporary insufficiency of the market, the government needs to take additional measures to promote the low-carbon upgrade of the coal power enterprises, which include penalties for excessive emissions and the incentive policy represented by low-carbon technology subsidies [16,17,18]. Therefore, it is necessary to explore the evolutionary path and equilibrium state of the interaction between the government and the coal power plants. The roles of the carbon trading scheme and power marketization in the process also need to be examined.

Many studies have been conducted on the low-carbon upgrade of the coal power plants, mainly including the elimination of the backward installation capacity and the adoption of decarbonization technologies [19,20]. On this basis, the feasibility of these upgrading paths and the associated influencing factors were evaluated. The structural optimization of the coal power industry is similar to the supply-side reform of the coal industry, which is characterized by the inferior elimination mechanism of the free market [21,22]. Such measures were supplemented by necessary policies and regulations which can promote the diffusion of modern coal-fired power generation technology and the natural elimination of the backward generation units. For ultra-supercritical coal-fired power units with high generation efficiency, utilizing the CCUS equipment was the preferred option to achieve the target of emission reduction, the diffusion of which was hindered by the cost factors [23]. The carbon costs saved by this device cannot cover its high R&D and operation costs. In addition, the high initial investment risk and the lack of supporting policies also limited its application in the coal-fired power industry [24,25]. The operation of the carbon market provided a new opportunity for the diffusion of the CCUS [26]. Morris (2019) found that when the carbon price reached $35–40, the coal power units with CCUS will get a cost advantage over the traditional ones; when the carbon price reaches $100, CCUS will achieve large-scale diffusion [12]. Compared with the carbon trading scheme, few research studies have taken the background of electricity marketization reform into consideration when studying the low-carbon transition of coal power in China. Even fewer have studied the integrated impacts of the carbon trading scheme and electricity marketization on the low-carbon development of coal power plants.

As the policy-maker of “carbon peaking and carbon neutrality”, the government of China is playing the leading role in guiding the domestic low-carbon transition and emission reduction. At the same time, it also maintains the interactive relationship with the carbon-intensive industries, which conforms to the research scope of evolutionary game theory [27,28]. The evolutionary game theory proposed by Taylor and Jonker (1978) and Abrams (2006) is based on the convergence process constituted by the evolutionary stability strategy and imitator dynamics [29]. Compared with the traditional game method, evolutionary game theory can perform the dynamic evolution process of the system through the study of the interaction mechanism of the related participants [30]. Evolutionary game theory has been widely applied to the research of carbon reduction between the government and other subjects including the high carbon-intensive industries, emission enterprises and consumers [31]. The partially rational subjects will change their decision dynamically under the goal of ensuring their maximum interests by observing and learning from each other [32]. The equilibrium state between the government and the implementers of emission reduction was highly dependent on the timing, intensity and duration of the intervention policy, which included regulation [33,34], penalties [35,36] and incentives [37,38], and the external factors such as progress in low-carbon technology and the development level of the carbon trading scheme. Additionally, evolutionary game theory was also used to analyze the interaction mechanism between the upstream and downstream enterprises for the low-carbon upgrade of the supply chain [39,40]. In terms of the low-carbon transition of the power sector, most of the existing studies focused on the large-scale development of renewable power and its substitution for coal power [7]. On this basis, the long-term impact of the support policy for renewable energy, which is represented by the quota system, on the low-carbon upgrading decision of coal power plants was analyzed. Considering the importance of coal power to the energy security of China, the research on the behavior characteristics of the government and the coal power enterprises from a market-oriented perspective, which is also helpful in formulating the leading policy for its low-carbon upgrade, needs to be supplemented.

On this basis, this study adopted evolutionary game theory to analyze the interaction mechanism, equilibrium state and the evolutionary path between the government and the coal power plants. Additionally, the impacts of the key factors, including the carbon trading scheme and the electricity market and differences among them, were further analyzed. The novelty of this work was mainly embodied in the evolutionary game model which integrated the subjects of the government and the coal power plants, as well as the external environment of carbon trading and electricity marketization. Based on this model, the potential effects of the carbon reduction policies, such as carbon quota, on the low-carbon upgrade of the domestic coal power can be evaluated. The research results can provide a decision-making basis for the coal power enterprises to achieve the goals of low-carbon transition and high-quality development. Additionally, this study can also provide practical suggestions for the government to guide the development of the domestic carbon market and the reform of electricity marketization for the strategic goals of carbon neutrality.

The remainder of this paper is organized as follows. Section 2 establishes the evolutionary game model between the government and the coal power enterprises. On this basis, the evolutionary game analysis is conducted in Section 3. The numerical analytical results are derived in Section 4. Further discussion and conclusion are provided in Section 5.

## 2. Evolutionary Game Model Construction

To urge the coal power plants to carry out the low-carbon transition, the government may take measures including emission verification and penalties for excess emissions and subsidies to encourage the adoption of low-carbon technology. In contrast, no action will be taken if the intervention costs surpass the expected earnings. Meanwhile, the profit-driven coal power plants will take the factors including technical level, carbon policy and government intervention into account before taking the decision on whether to adopt the CCUS technology [20,41]. On this basis, an evolutionary game model was established to describe the interaction between the two parties. An equilibrium analysis was conducted based on the theoretical model. After that, a numerical analysis was further applied to explore how the change of critical parameters can influence the equilibrium states. Due to the volatility and unpredictability of some factors such as power production and carbon prices, obtaining the precise values of parameters was impractical. Therefore, on the premise of ensuring the logic of their relative sizes, the initial values of the parameters were set based on the existing literature.

### 2.1. Model Assumptions

**H1.**  *If the government chooses not to intervene in the low-carbon transition of the coal power plants, it only needs to assume the environmental costs caused by the excessive carbon emissions. On the contrary, the government will expend extra costs to verify and supervise the carbon emissions and provide subsidies for the coal power plants adopting the CCUS technology.*

**H2.**  *In the carbon market, the government will adjust the quota policy according to the goal of emission reduction and the capability of the coal power industry. In contrast, the coal power enterprises can sell the remaining quota in the carbon market when their actual carbon emissions are lower than the allocated amount. Conversely, when the emissions exceed the quota, they need to buy extra emission rights from other participants.*

**H3.**  *With the development of the carbon trading market and the promotion of electricity marketization, the “pioneers” who adopt the CCUS first will gain more power generation opportunities due to two reasons. On the one hand, power plants with CCUS can largely reduce the carbon emission cost from generation, thus gaining a cost advantage over those without CCUS [41,42]. On the other hand, thermal power plants in China are mostly state-owned and at the command of government. The government has imposed restrictions on the production of high-emission plants for environmental conservation, especially in recent years under the goals of carbon peak and carbon neutrality [43]. On this basis, this study used the indicator* v *to represent such positive effect of the upgrading decision (* v>1 *). On the contrary, the coal power plants that choose not to upgrade will become less competitive in the electricity market. Another indicator,* w *, was used to represent the reduction of generation opportunities of the power plants maintaining the original generation mode (* w<1 *).*

On this basis, Table 1 shows the details of all indicators used in the game model.

### 2.2. Model Construction

In our 2×2 evolutionary game model, the government can make stochastic and independent decisions between intervention and non-intervention, and the coal power plants between the low-carbon upgrade and maintenance. Table 2 shows the expected payoff matrix of the two stakeholders.

x was the probability that the government chose to intervene in the low-carbon transformation of coal power plants, and 1−x was the probability that the government chooses not to intervene. Correspondingly, y was the probability that the coal power plants chose to install the CCUS device, and 1−y was the probability that the coal power plants chose not to change. The government and coal power enterprises in the game were self-interested rational actors. After long-term interaction and adjustment of their own strategies, the evolutionarily stable strategy (ESS) can be achieved.

## 3. Evolutionary Game Analysis

### 3.1. Evolutionary Stability Analysis

First, the average expected return of the two parties in the evolutionary game were calculated. $U_{g_{1}}$ and $U_{g_{2}}$ represented the expected return of the government by choosing the strategies of “intervention” and “non-intervention”, respectively, while $\bar{U_{g}}$ was the average return; all were described as follows:

(1) $$\begin{array}{c} U_{g_{1}}=y\left(-o_{2}-j-s+∆q\right)+\left(1-y\right)\left(-o_{1}-j+c\right) \end{array}$$

(2) $$\begin{array}{c} U_{g_{2}}=y\left(-o_{2}\right)+\left(1-y\right)\left(-o_{1}\right) \end{array}$$

(3) $$\begin{array}{c} \bar{U_{g}}=xU_{g_{1}}+\left(1-x\right)U_{g_{2}} \end{array}$$

Similarly, $U_{t_{1}}$ and $U_{t_{2}}$ denoted the expected return of the coal power plants on the strategies of “low-carbon upgrade” and “maintenance”, respectively, and $U_{t}$ was the average return. They were described as follows:

(4) $$\begin{array}{c} U_{t_{1}}=x\left(v\left(r-c_{2}-p\left(e_{2}-E\right)\right)+s-k\right)+\left(1-x\right)\left(v\left(r-c_{2}-p\left(e_{2}-E\right)\right)-k\right) \end{array}$$

(5) $$\begin{array}{c} U_{t_{2}}=x\left(w\left(r-c_{1}-p\left(e_{1}-E\right)\right)-c\right)+\left(1-x\right)\left(r-c_{1}-p\left(e_{1}-E\right)\right) \end{array}$$

(6) $$\begin{array}{c} \bar{U_{g}}=yU_{t_{1}}+\left(1-y\right)U_{t_{2}} \end{array}$$

Accordingly, based on the method proposed by Taylor and Jonker [44], the replicator equations of the government and the coal power plants were formulated as Equations (7) and (8), respectively.

(7) $$\begin{array}{c} F\left(x\right)=\frac{dx}{dt}=x\left(U_{g_{1}}-\bar{U_{g}}\right)=x\left(1-x\right)\left(-sy-cy-j+c+y∆q\right) \end{array}$$

(8) $$\begin{array}{c} \;\;\;\;\;\;\;\;\;\begin{array}{c} F\left(y\right)=\frac{dy}{dt}=y\left(U_{t_{1}}-\bar{U_{t}}\right)=y\left(1-y\right)(-rwx+e_{1}pwx-Epwx+c_{1}wx+cx+\\ sx+rx-e_{1}px+Epx-c_{1}x+rv-e_{2}pv+Epv-k-c_{2}v-r+e_{1}p-Ep+c_{1}) \end{array} \end{array}$$

Equations (7) and (8) together constituted the replicated dynamic system and local equilibrium point (LEP), which can be achieved by letting Equation (7) =0 and Equation (8) =0 [45]. Thus, we can get five LEPs of the system as follows: (0, 0), (0, 1), (1, 0), (1, 1) and ($X_{P}$, $Y_{P}$), $x\in \left[0,\;1\right]$, $y\in \left[0,\;1\right]$, where $X_{P}=\frac{-rv+e_{2}pv-Epv+k+c_{2}v+r-e_{1}p+Ep-c_{1}}{-rw+e_{1}pw-Epw+c_{1}w+c+s+r-e_{1}p+Ep-c_{1}}$, $Y_{P}=\frac{c-j}{c+s-∆q}$.

As the above LEPs were not necessarily the ESS, it was necessary to examine their stability through the Jacobian matrix of the system. The partial derivative with respect to x and y were taken into Equations (7) and (8) and then the Jacobian matrix was obtained as follows:

(9) $$\begin{array}{c} \;J=\left(\begin{array}{cc} a_{11} & a_{12}\\ a_{21} & a_{22} \end{array}\right)\;\;\;\;\;\;\;\;\;\;\;\;\;\;\;\;\;\;\;\;\;\;\;\;\;\;\;\;\;\;\;\;\;\;\;\;\;\;\;\;\;\;\;\;\;\;\;\;\;\;\;\;\;\;\;\;\;\;\;\;\;\;\;\;\;\;\;\;\;\;\;\;\;\;\;\;\;\;\;\;\;\;\;\;\;\;\;\;\;\;\;\;\;\;\;\;\;\;\;\;\;\;\;\\ \;\;\;\;\;\;\;\;\;\;=\left(\begin{array}{cc} \left(1-2x\right)\left(-sy-cy-j+c+y∆q\right) & x\left(1-x\right)\left(-s-c+∆q\right)\\ y\left(1-y\right)f_{1} & \left(1-2y\right)f_{2} \end{array}\right) \end{array}$$

where $f_{1}=-rw+e_{1}pw-Epw+c_{1}w+c+s+r-e_{1}p+Ep-c_{1}$, $f_{2}=-rwx+e_{1}pwx-Epwx+c_{1}wx+cx+sx+rx-e_{1}px+Epx-c_{1}x+rv-e_{2}pv+Epv-k-c_{2}v-r+e_{1}p-Ep+c_{1}$, $f_{3}=rv-e_{2}pv+Epv-k-c_{2}v-r+e_{1}p-Ep+c_{1}$, and $f_{4}=-rw+e_{1}pw-Epw+c_{1}w+c+s+rv-e_{2}pv+Epv-k-c_{2}v$.

After plugging the LEP values as $\left(x,y\right)$ into the Jacobian matrix, the determinant (*det*) and trace (*tr*) of each Jacobian matrix can be obtained, as shown in Table 3. The corresponding LEP is evolutionarily stable when the conditions of det J>0 and tr J<0 can be met [45].

To simplify the calculation process, three indicators were added. $k^{\prime }$ denoted the transition costs of the power plants under the government’s decision for “intervention”, and $R_{2}$ and $R_{1}$ denoted the profits of the coal power plants from selling electricity by choosing or choosing not to adopt the CCUS, respectively. Where $k^{\prime }=k-s-c\;(k^{\prime }<k)$, $R_{1}=r-c_{1}-p\left(e_{1}-E\right)$ and $R_{2}=r-c_{2}-p\left(e_{2}-E\right)$. Thus, the relative size of $R_{1}$ and $R_{2}$ was determined by the related factors including the carbon price and the technological level of the CCUS.

### 3.2. Equilibrium Analysis

(1) When c<j and $vR_{2}-R_{1}<k$, the LEP of the system was (0, 0), namely non-intervention and maintenance. Here the earnings of the government from charging the carbon-intensive plants was lower than the regulatory costs, and the sum of the carbon costs saved by using the CCUS and profits brought by the increased generation opportunities cannot cover the upgrading costs of the enterprises. In this case, even if the coal power enterprises tended to adopt the CCUS at first, they will eventually quit, as the upgrading costs are unaffordable without the financial assistance from the government. Besides, the high costs of the regulatory activities, including the supervision and inspection of carbon emissions, reduced the willingness of the government to implement interventions.

(2) When ∆q<s+j and $vR_{2}-R_{1}>k$, the LEP of the system was (0, 1), namely non-intervention and low-carbon upgrade). Here the environmental costs saved by the government’s subsidies cannot offset its subsidies and regulatory costs, and the profits from adopting the CCUS exceed its upgrading costs. In this case, the government will rapidly terminate the subsidies and regulation. Due to the decrease in the upgrading cost, the CCUS technology became attractive to the coal power plants even when financial subsidies were absent. The technological progress will promote the low-carbon transition of coal power. When the costs of the CCUS were close or even lower than the expected profits from it, coal power plants will spontaneously make the upgrading decision. At the same time, the cost of encouraging the low-carbon upgrade can be saved by the government. Therefore, this equilibrium state was the ideal plan for the low-carbon transition of coal power.

(3) When c>j and $vR_{2}-wR_{1}<k^{\prime }$, the LEP of the system was (1, 0), namely intervention and maintenance. Considering the low carbon price and insufficient marketization of electricity, and the high technical cost, the net revenue of the upgrade strategy for the coal power enterprises was negative. In this case, the decision for maintenance was still preferred under the punishment mechanism for the excess emissions. The subsidy policy was not working when the external environment could not guarantee the ideal profits for the coal power plants using the CCUS. Therefore, a functional carbon market and electricity market should be established in the early stage of the low-carbon electricity transition. Moreover, a comprehensive system for inspecting and supervising the carbon emissions was also needed to ensure the government can regulate the coal power plants at a lower cost.

(4) When ∆q>s+j and $vR_{2}-wR_{1}>k^{\prime }$, the LEP of the system was (1, 1), namely intervention and low-carbon upgrade. Here the total cost of the government from regulation and subsidies was lower than the negative external cost saved, and the CCUS could bring a net positive profit to the coal power plants. In this case, the government will actively intervene in the coal power transition and offer considerable subsidies, which promote the low-carbon transition of coal power. Additionally, the upgrade of the coal power plants will give positive feedback to the government in terms of emission reduction and environmental benefits, and then a virtuous circle can be formed.

(5) When g<0, the LEP of the system was ($X_{P}$, $Y_{P}$). It was a hybrid-strategy Nash equilibrium, where the government and coal power plants chose their strategies with a certain probability of obtaining the maximum expected return. In this case, the strategy combination of the government and coal power plants fluctuated periodically around ($X_{P}$, $Y_{P}$).

On this basis, the dynamic evolution of the game between the government and coal power enterprises can be described as follows. The government needs to assume the costs of the supporting measures for emission verification and supervision in the early stage of the carbon trading, which directly increases the cost for implementing intervention. Meanwhile, due to the high emission quota and low carbon price, the profits of power enterprises from adopting the CCUS cannot cover the associated costs. The equilibrium of the game is thereby (non-intervention, maintenance). With the accumulation of experience and the improvement of the auxiliary system, the cost of the government regulation will gradually decline, and the negative external costs saved by the subsidies will exceed the sum of the financial subsidies and regulatory costs. With the increase of the carbon price and technological development of CCUS, coal power plants will gradually make the upgrade decision. A new equilibrium of intervention and low-carbon upgrade can be reached. With the proceeding of the low-carbon coal power transition, the effect of the government’s intervention strategy is decreasing. Eventually, the coal power plants will adopt the CCUS technology spontaneously in the circumstances of a mature carbon and electricity market and a high technological level. As a result, an equilibrium of non-intervention and low-carbon upgrade will be in place. In addition, due to the complexity and dynamics of the external environment, there may be short-term periodic fluctuations between two equilibrium states.

## 4. Evolutionary Simulation Analysis

The ideal equilibrium state of the game was that the coal power enterprises spontaneously adopted the CCUS under the pressure of carbon costs in a sound market mechanism without additional intervention from the government, which was consistent with the LEP (0, 1). Considering that the evolutionary path and the equilibrium state were determined by the internal and external environment of the game, the simulation analysis was conducted to identify the importance of related factors. Based on the payoff matrix, three types of the factors were distinguished, namely changes of generation opportunities, technological level and carbon trading scheme.

The values of the parameters were defined based on a 600 MW coal power plant with a lifetime of 40 years. The carbon intensity without CCUS is 0.78 t CO_2_/MWh, and the utilization of CCUS can reduce 90% of the carbon dioxide emission [46]. The coal cost per unit of power generation in China was estimated at $0.033/kWh and the on-grid price of coal-fired power was around $0.064/kWh [47]; hence, the fuel cost $c_{1}$ took up about 50% of the power generation revenue r. The adoption of CCUS will impose an electricity penalty on the production and thus consume more coal per unit of power generation. The penalty rate was dependent on power generation scale, generation technology and capture method [48,49]. The technical and cost requirements were positively related to the capture rate. In this paper, an intermediate value of 50% was used and the corresponding penalty rate was around 6% [50]. Therefore, $c_{1}$ increased by 6%. On this basis, the relative values of the parameters were derived. Due to the ideal equilibrium state of (non-intervention, low-carbon upgrade) being a future scenario, accurate values of some indicators was missing, such as  ∆q and *j*. In this case, the values of such paraments were deduced based on field research [51,52] and the corresponding constraint conditions of ∆q<s+j and $v\left(R_{2}\right)-R_{1}>k$, where $R_{1}=r-c_{1}-p\left(e_{1}-E\right)$ and $R_{2}=r-c_{2}-p\left(e_{2}-E\right)$.

On this basis the initial values of the parameters were set as follows: $c=100,\;j=50,\;∆q=120,\;s=80,\;r=1000,\;w=0.95,\;v=1.1,\;c_{1}=500,\;c_{2}=530,\;p=50,\;e_{1}=10,\;e_{2}=5,\;E=7\;\mathrm{and}\;k=250$. The initial strategy probability of the two parties was set as (0.5,0.5).

### 4.1. Changes of Generation Opportunity

#### 4.1.1. Increase of Generation Opportunity by *v*

**Figure 1:**
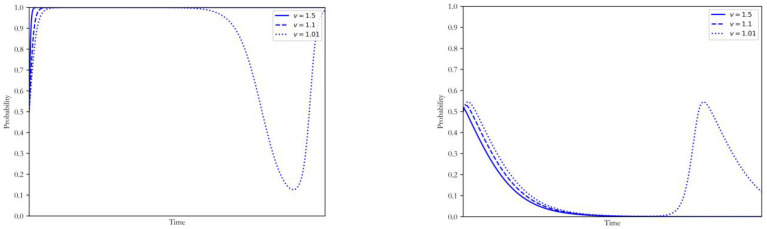
Influence of changing v on the strategy of coal power plants and government.

Maintaining other factors unchanged, the increasing degree of the generation opportunity from the adoption of the CCUS v was taken as 1.5, 1.1 and 1.05, respectively. The simulation results of strategy selection by both sides of the game are shown in Figure 1.

The revenue of selling electricity from the additional generation opportunities can significantly stimulate the low-carbon upgrade of the coal power plants. As a result, the decision of the power plants will rapidly be restrained to the ESS of upgrade, and this process will be accelerated with the increase of v. Accordingly, the government can achieve the strategic goal of low-carbon coal power transition without additional regulatory measures and subsidy costs. The government will be restrained to the ESS of non-intervention, the speed of which is also accelerated with the increase of v. However, when the earnings from the additional generation opportunities were limited by the developing carbon trading scheme and low level of electricity marketization, the intervention by the government was still needed. In this case, the game process will present an unstable state where the coal power plants and the government will constantly change their strategies before reaching the new long-term equilibrium.

#### 4.1.2. Decrease of Generation Opportunity by *w*

**Figure 2:**
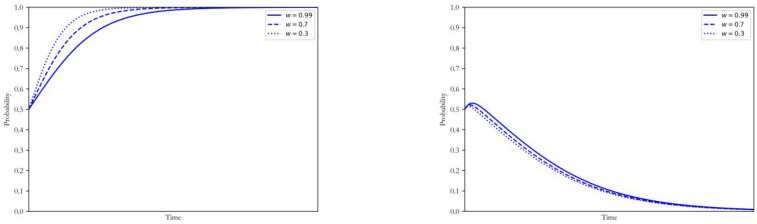
Influence of changing w on the strategy of coal power plants and government.

With other parameters unchanged, the decreasing degree of generation opportunity (w) for the enterprises maintaining the original generation mode was taken as 0.99, 0.7 and 0.5, respectively. The simulation results of strategy selection by both sides of the game were shown in Figure 2.

The profitability of coal power plants giving up the upgrading opportunity would be further compressed in the carbon trading environment and this would put them at a disadvantage in the market competition. In that case, they were motivated to implement the CCUS technology. Specifically, with the decrease of *w*, the coal power enterprises would be restrained to the low-carbon upgrading decision more quickly. The effect of the government’s intervention was limited in this case. Therefore, the government tended to gradually reduce its intervention intention. The size of w was determined by the relative generation costs of coal power enterprises with or without the CCUS as well as the level of electricity marketization. Therefore, the government can not only deepen the power marketization reform while ensuring the stable development of the carbon market to reduce the competitiveness of traditional coal power plants, but also limit the proportion of the carbon-intensive electricity on the grid through the implementation of the supply-side reform. These measures were useful in encouraging the coal power enterprises to carry out the low-carbon transition and eliminate the backward installed capacity.

### 4.2. Technological Level

#### 4.2.1. Acquisition Cost *k*

**Figure 3:**
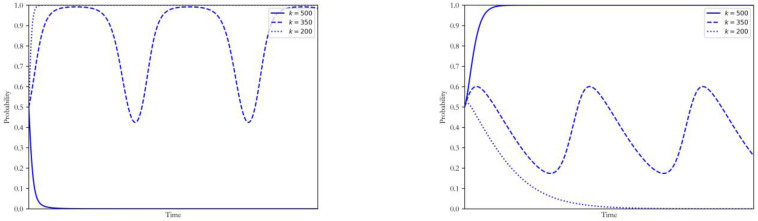
Influence of changing k on the strategy of coal power plants and government.

The CCUS can capture more than 90% of the carbon dioxide emissions from the power plants, and the high cost is the main obstacle to its diffusion at present. On this basis, this section mainly discusses how the change of acquisition cost k and generation cost c, which can be improved by technological progress and learning effect, can influence the equilibrium states of the game. Other factors remaining unchanged, the acquisition cost k was taken as 500, 350 and 200, respectively. The simulation results of strategy selection by both sides of the game were shown in Figure 3.

When the acquisition cost of the CCUS (k=500) was significantly higher than the penalty cost for the excess carbon emissions, the coal power plants will quickly be restrained to the decision for maintenance. In contrast, the corresponding benefit from penalising carbon-intensive enterprises was higher than the regulatory expenditure, and the government tends to conduct the intervention. With the development of low-carbon technology, the acquisition cost was supposed to decrease (k=350). In that case, coal power plants and the government will constantly adjust their decisions according to each other’s strategies. Specifically, when the government intervention was sensitive, the power plants can make up for the upgrading costs by the transition benefits and the government’s subsidies, and then enhance their willingness to adopt the CCUS. On the other hand, when the government was less proactive, the power plants received fewer financial subsidies and penalties, which resulted in the decision for maintenance. When this technology is mature (k=200), coal power plants can largely save the costs from emission with low investment. In that case, coal power plants will adopt the CCUS immediately without the government intervention, and the government will simultaneously be restrained to a non-intervention decision.

#### 4.2.2. Generation Cost $c_{2}$

**Figure 4:**
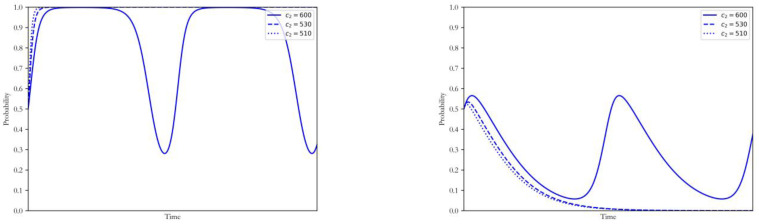
Influence of changing $c_{2\;}$ on the strategy of coal power plants and government.

With other parameters remaining unchanged, the generation cost $c_{2\;}$ is taken as 600, 530 and 510, respectively. The simulation results of strategy selection by both sides of the game are shown in Figure 4.

The impacts of the change of generation cost on the strategies of coal power enterprises and the government were similar to the case of the acquisition cost. When the generation efficiency decreased greatly after the upgrade, its benefits could not fully compensate for the increase in fuel cost. In that case, the strategy of the coal power plants was dependent on the degree of government intervention, and it was hard to reach a pure-strategy equilibrium. When the generation efficiency decreased slightly after adopting the CCUS ($c_{2\;}=510\;\mathrm{or}\;530$), the benefits from reducing carbon cost could make up for the increase in generation cost. In that case, the strategy of the government cannot change power plants’ decision for a low-carbon upgrade. When the government implemented intense regulation and subsidies, the willingness of coal power plants to conduct the low-carbon upgrade would be strengthened. In contrast, the additional generation costs could not be fully covered when the intervention from the government was weak, which resulted in low motivation for the enterprises to conduct the upgrade.

The technological level of the CCUS had a significant impact on the equilibrium state of the game between the government and coal power enterprises. With the development of technology and the accumulation of learning effects, the cost for coal power plants to adopt CCUS technology will continue declining. In that case, the implementation of the government subsidies, penalties and other regulatory measures can promote the low carbon upgrading of the coal power enterprises. When the CCUS technology is advanced, the energy loss from it will be significantly reduced [53,54]. Under the carbon trading market, the traditional coal power plants with high carbon intensity will tend to upgrade naturally, and this result will not change without the government’s intervention.

### 4.3. Carbon Trading Scheme

#### 4.3.1. Carbon Price p

**Figure 5:**
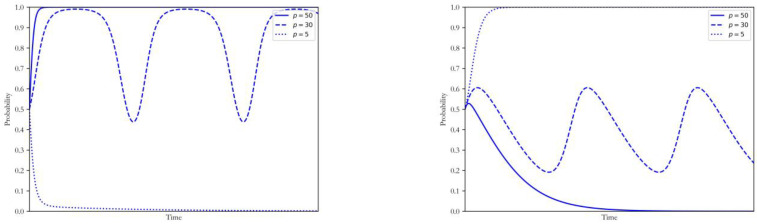
Influence of changing p on the strategy of coal power plants and government.

With other parameters unchanged, the carbon price p was taken as 50, 30 and 5, respectively. The simulation results of both sides of the game are shown in Figure 5. The coal power plants will quickly be restrained to the strategy of maintenance at the establishment stage of the carbon market where the carbon price was low. In contrast, the government tended not to intervene in the transition process. With the increase of the carbon price, coal power plants and the government will constantly change their own strategies according to each other’s decisions, forming a fluctuating state around ($X_{P}$, $Y_{P}$). When the carbon price was at a high level, coal power plants will reach the ESS of upgrading the CCUS.

As the most important tool for emission reduction, the carbon trading scheme played a vital role in promoting the low-carbon upgrade of the coal power enterprises. When the carbon price was low, the necessity and urgency of low carbon upgrading for coal power plants were insufficient. With the development of a carbon market and increase in carbon price, power plants will make flexible adjustments to their strategy according to the degree of governmental intervention. When the carbon market mechanism is relatively developed and the carbon price is high, they will be forced to upgrade their units to reduce the carbon costs, even without any government incentives. At present, the relevant departments should continue developing the domestic carbon trading scheme to realize the low-carbon coal power transition from the government-leading pattern to that of the market-oriented pattern.

#### 4.3.2. Emission Quota E

**Figure 6:**
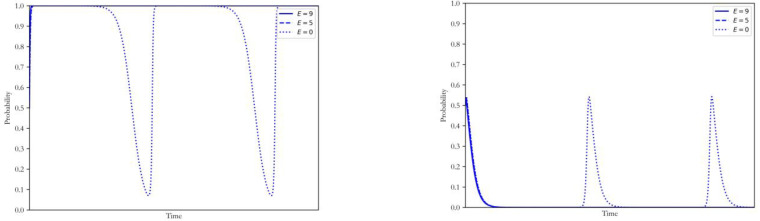
Influence of changing E on the strategy of coal power plants and government.

Other factors remaining unchanged, the technology cost E was taken as 9, 5, and 0, respectively, which corresponded to 90%, 50% and 0% of the total carbon emissions of coal power plants. The simulation results of strategy selection by both sides of the game are shown in Figure 6.

It can be found that when the carbon quota proportion is low, the coal power plants and the government will reach a hybrid-strategy Nash equilibrium. In contrast, when the carbon quota proportion was high, the strategy of the coal power plants unexpectedly turned to adopting the CCUS, and the corresponding strategy of the government was non-intervention. This result indicated that the allocation of the quota should match the development level of the carbon market. In the early stage of the carbon market, the strict quota policy cannot lead to the low-carbon upgrade of the coal power plants. The reason for such a phenomenon was that the carbon trading scheme allowed the power enterprises with low carbon intensity to obtain additional revenue by selling excess quotas to those maintaining the original generation mode, which stimulated the emergence of forerunners of low-carbon upgrade. In addition, the existence of the market mechanism represented by the factors of v and w also made the low-carbon upgrade more profitable when the quota was high. It was noteworthy that such results did not mean that the quota system was ineffective for the low carbon transition of coal power, but that its impact was closely related to the stage of the low-carbon transition and the carbon market. Without adequate pre-research on the external environment, the adjustments of the quota were unlikely to have a satisfactory influence on the low-carbon transition. Moreover, the electricity market and other policy tools can also be used to improve the competitiveness of low-carbon coal power plants through the effects of v and w.

## 5. Conclusions

Based on the evolutionary game theory, this paper studied the interaction mechanism between the coal power enterprises and the government in the process of China’s low-carbon electricity transition. Five evolutionarily stable strategies and the corresponding conditions were identified and defined. On this basis, the dynamic evolutionary process between the government and the coal power enterprises were depicted from the equilibrium state of non- intervention and maintenance to that of non-intervention and low-carbon upgrade. There were two transitory stages, namely intervention and low-carbon upgrade and intervention and low-carbon upgrade, before the coal power enterprises could spontaneously make the upgrade decision driven by the development of the carbon trading scheme and formation of the pricing mechanism for the electricity market. Additionally, the evolutionary simulation analysis was conducted to explore the impacts of the influencing factors on the evolutionary equilibrium states.

The results showed that the fair competition environment of the domestic electricity market played a vital role in the low-carbon upgrading of coal power. With the introduction of the carbon trading scheme, the bidding mechanism of the electricity market can achieve the filtering function to promote the development of the low-carbon electricity [55,56]. In this study, the additional generation opportunity for the coal power plants with CCUS was measured by the indicator v. The increase of v can contribute to the diffusion of the CCUS among coal power plants, while the influence from the reduction of power generation opportunities (w) is less significant. The high cost of the CCUS was the major obstacle for the low-carbon upgrade of the coal power plants. The system will quickly be restrained to the ideal state (non-intervention, upgrade) when the technological cost is largely reduced. This result was consistent with the results of the researches on the investment decision of CCUS [20,41] that the cost was the determinative factor for its diffusion. The carbon cost can directly promote the low-carbon transition of coal power and exert an important impact on the final equilibrium state. In addition, under different carbon prices, the effect of government intervention varied as well, which led to the shift of the government’s strategy. A tighter allocation plan of an emission quota does not necessarily promote the application of the CCUS. Considering that the excess emission quota can be traded to obtain additional benefits, the slack quota policy can stimulate the emergence of the forerunners of a low-carbon upgrade, which was consistent with the finding of Wang (2019) [57] that higher free quotas contributed to an increase of the carbon reduction rate. The high operation costs for emission inspection and supervision will discourage the government from intervening in the low-carbon transition of coal power. With the establishment of the supporting system, the government tends to actively conduct the intervention until the low-carbon transition of coal power can be achieved through the mature carbon and electricity market mechanisms, and then it will be restrained to the “non-intervention” strategy eventually.

In order to promote the domestic low-carbon transition of coal power, this study put forward the following suggestions based on the research results:

(1) The reform of the electricity marketization should be continued to promote the formation of a pricing mechanism, which is helpful for realizing the impact of the carbon trading scheme on carbon reduction. In this way, the backward generation units can be eliminated naturally and more generation opportunities can be transferred to the low-carbon coal power plants adopting the CCUS.

(2) The preferred disbursement of government subsidies should be shifted from the acquisition of the decarbonization equipment to the R&D investment. In this way the competitiveness of the CCUS can be improved fundamentally, which can significantly contribute to its large-scale diffusion in domestic coal power enterprises.

(3) A comprehensive supporting system of carbon trading should be established to improve the government’s ability to implement carbon inspection and supervision at lower cost and simultaneously guide the coal power enterprises to conduct the low-carbon transition at the initial stage of the carbon market.

Several further aspects remain outside the scope of this study. As another important participant of the low-carbon electricity transition, renewable power enterprises can be included into the theoretical framework. Based on the mechanism of the tripartite game, more details of the transition process can be depicted. In addition, the decision model of the power enterprises which internalizes the related parameters and takes the effects of uncertainty into consideration can be established, to promote the practical contribution of this study.

## Figures and Tables

**Table 1:** Parameter symbols and meanings.

Indicator	Description	Notes
$N\equiv \left(g,k\right)$	Set of the participants	g and k represent the government and coal power enterprises, respectively
$Z_{g}=\left\{S,NS\right\}$	Set of the government decisions	S represents intervention and NS represents non-intervention
$Z_{k}=\left\{U,NU\right\}$	Set of the coal power plant decisions	U represents low-carbon upgrade and NU represents maintenance
s	Subsidies to the coal power plants	s>0
j	Costs for the regulatory activities	j>0
c	Punishment for the excessive carbon emission	c>0
k	Upgrading costs of the CCUS	k>0
$c_{1},c_{2}$	The generation cost before and after adopting the CCUS	$c_{2}>c_{1}>0$
r	Initial revenue of coal power plants from selling electricity	r>0
$e_{1},\;e_{2}$	Carbon emission rates before and after using CCUS	$e_{1}>e_{2}>0$
$o_{1},\;o_{2}$	Negative external costs caused by carbon emission before and after using CCUS	$o_{1}>o_{2}>0$
∆q	Changes of the negative external costs by the government subsidies	∆q>0
E	Carbon quota	E>0
p	Carbon price	p>0
v	Increase of generation opportunity	v>1
w	Decrease of generation opportunity	w<1

**Table 2:** The payoff matrix between the government and coal power enterprises.

		Coal Power Enterprises	
		Low-Carbon Upgrade (*U*)	Maintenance (*NU)*
Government	Intervention (*S*)	$-o_{2}-j-s+∆q$, $v\left[r-c_{2}-p\left(e_{2}-E\right)\right]+s-k$	$-o_{1}-j+c$, $w\left[r-c_{1}-p\left(e_{1}-E\right)\right]-c$
	Non-intervention (*NS*)	$-o_{2}$, $v\left[r-c_{2}-p\left(e_{2}-E\right)\right]-k$	$-o_{1}$, $r-c_{1}-p\left(e_{1}-E\right)$

**Table 3:** *det J* and tr J of the Jacobian matrix *J* for each LEP.

LEP	det J	tr J
(0, 0)	$\left(c-j\right)\;\left(vR_{2}-R_{1}-k\right)$	$vR_{2}-R_{1}-k+\left(c-j\right)$
(0, 1)	$\left(∆q-s-j\right)\left(-vR_{2}+R_{1}+k\right)$	$R_{1}-vR_{2}+k+\left(∆q-s-j\right)$
(1, 0)	$\left(-c+j\right)\left(vR_{2}-wR_{1}-\;k\prime \right)$	$vR_{2}-wR_{1}+s+c-k-\left(c-j\right)$
(1, 1)	$\left(-∆q+s+j\right)\left(-vR_{2}+wR_{1}+\;k\prime \right)$	$wR_{1}-vR_{2}-s-c+k-\left(∆q-s-j\right)$
$(X_{P}$$,\;Y_{P}$)	0	g

Note: $g=\frac{∆q\left(c-j\right)\left(2v\left[r-c_{2}-p\left(e-E\right)\right]-w\left[r-c_{1}-p\left(e_{1}-E\right)\right]-\left[r-c_{1}-p\left(e_{1}-E\right)\right]-2k+s+c\right)}{\left(c+s-∆q\right)\left(w\left[r-c_{1}-p\left(e_{1}-E\right)\right]-\left[r-c_{1}-p\left(e_{1}-E\right)\right]-s-c\right)}$.

## Data Availability

Not applicable.

## References

[1] IEA Key World Energy Statistics. https://www.iea.org/statistics/.

[2] Relva S.G., Silva V., Gimenes A., Udaeta M., Peyerl D. (2021). Enhancing developing countries’ transition to a low-carbon electricity sector. Energy.

[3] Zhang S., Andrews-Speed P., Li S. (2018). To what extent will China’s ongoing electricity market reforms assist the integration of renewable energy?. Energy Policy.

[4] Members R.E.N. (2022). Renewables 2022 Global Status Report 2022.

[5] Shivashankar S., Mekhilef S., Mokhlis H., Karimi M. (2016). Mitigating methods of power fluctuation of photovoltaic (PV) sources–A review. Renew. Sustain. Energy Rev..

[6] Gazheli A., van den Bergh J. (2018). Real options analysis of investment in solar vs. wind energy: Diversification strategies under uncertain prices and costs. Renew. Sustain. Energy Rev..

[7] Yao X., Yi B., Yu Y., Fan Y., Zhu L. (2020). Economic analysis of grid integration of variable solar and wind power with conventional power system. Appl. Energy.

[8] National Development and Reform Commission of China China 2050 High Renewable Energy Penetration Scenario and Roadmap Study. https://www.efchina.org/Reports-en/china-2050-high-renewable-energy-penetration-scenario-and-roadmap-study-en.

[9] Umar M., Riaz Y., Yousaf I. (2022). Impact of Russian-Ukraine war on clean energy, conventional energy, and metal markets: Evidence from event study approach. Resour. Policy.

[10] Steffen B., Patt A. (2022). A historical turning point? Early evidence on how the Russia-Ukraine war changes public support for clean energy policies. Energy Res. Soc. Sci..

[11] Chen Q., Kang C., Xia Q., Kirschen D.S. (2012). Optimal flexible operation of a CO_2_ capture power plant in a combined energy and carbon emission market. IEEE Trans. Power Syst..

[12] Morris J., Paltsev S., Ku A.Y. (2019). Impacts of China’s emissions trading schemes on deployment of power generation with carbon capture and storage. Energy Econ..

[13] Li M., Gao H., Abdulla A., Shan R., Gao S. (2022). Combined effects of carbon pricing and power market reform on CO2 emissions reduction in China’s electricity sector. Energy.

[14] Shen J., Cheng C., Jia Z., Zhang Y., Lv Q., Cai H., Wang B., Xie M. (2022). Impacts, challenges and suggestions of the electricity market for hydro-dominated power systems in China. Renew. Energy.

[15] Pollitt M.G. (2021). Measuring the impact of electricity market reform in a Chinese context. Energy Clim. Chang..

[16] He Y., Guo S., Dong P., Huang J., Zhou J. (2023). Hierarchical optimization of policy and design for standalone hybrid power systems considering lifecycle carbon reduction subsidy. Energy.

[17] Shao C., Ding Y., Wang J. (2019). A low-carbon economic dispatch model incorporated with consumption-side emission penalty scheme. Appl. Energy.

[18] Kruse-Andersen P.K., Sørensen P.B. (2022). Optimal energy taxes and subsidies under a cost-effective unilateral climate policy: Addressing carbon leakage. Energy Econ..

[19] Zhang M.M., Wang Q., Zhou D., Ding H. (2019). Evaluating uncertain investment decisions in low-carbon transition toward renewable energy. Appl. Energy.

[20] Chen H., Wang C., Ye M. (2016). An uncertainty analysis of subsidy for carbon capture and storage (CCS) retrofitting investment in China’s coal power plants using a real-options approach. J. Clean. Prod..

[21] Zhang Y., Nie R., Shi R., Zhang M. (2018). Measuring the capacity utilization of the coal sector and its decoupling with economic growth in China’s supply-side reform. Resour. Conserv. Recycl..

[22] Shi X., Rioux B., Galkin P. (2018). Unintended consequences of China’s coal capacity cut policy. Energy Policy.

[23] Størset S.Ø., Tangen G., Berstad D., Eliasson P., Hoff K.A., Langørgen Ø., Munkejord S.T., Roussanaly S., Torsæter M. (2019). Profiting from CCS innovations: A study to measure potential value creation from CCS research and development. Int. J. Greenh. Gas Control.

[24] Xiao K., Yu B., Cheng L., Li F., Fang D. (2022). The effects of CCUS combined with renewable energy penetration under the carbon peak by an SD-CGE model: Evidence from China. Appl. Energy.

[25] Kawai E., Ozawa A., Leibowicz B.D. (2022). Role of carbon capture and utilization (CCU) for decarbonization of industrial sector: A case study of Japan. Appl. Energy.

[26] Fan J.-L., Xu M., Wei S.-J., Zhong P., Zhang X., Yang Y., Wang H. (2018). Evaluating the effect of a subsidy policy on carbon capture and storage (CCS) investment decision-making in China—A perspective based on the 45Q tax credit. Energy Procedia.

[27] You-Dong L.I., Xia L.J., Wang F.Z. (2019). Game and Coordination Model for Low-carbon Supply Chain with Product Substitution. Chinese J. Manag. Sci..

[28] Jie X., Chen F.J., Liu G.P. (2021). Research on the Tripartite Evolutionary Game Model of Government, Enterprises and Travelers under the Carbon Tax Policy for Motor Vehicle. Oper. Res. Manag. Sci..

[29] Wang G., Chao Y., Cao Y., Jiang T., Han W., Chen Z. (2022). A comprehensive review of research works based on evolutionary game theory for sustainable energy development. Energy Rep..

[30] Zhou X., Jia M., Wang L., Sharma G.D., Zhao X., Ma X. (2022). Modelling and simulation of a four-group evolutionary game model for green innovation stakeholders: Contextual evidence in lens of sustainable development. Renew. Energy.

[31] Chu Z., Bian C., Yang J. (2022). Air Pollution Regulations in China: A Policy Simulation Approach with Evolutionary Game Przepisy dotyczące zanieczyszczenia powietrza w Chinach: Podejście do symulacji polityki i gry ewolucyjnej. Probl. Ekorozw..

[32] Friedman J.W., Mezzetti C. (2002). Bounded rationality, dynamic oligopoly, and conjectural variations. J. Econ. Behav. Organ..

[33] Qu G., Yang L., Qu W., Li Q. (2021). Game model to analyze strategy option between government regulation and public supervision in the third party international environmental audit. Chin. J. Manag. Sci..

[34] Wang Y., Xin X.U., Zhu Q. (2021). Carbon emission reduction decisions of supply chain members under cap-and-trade regulations: A differential game analysis. Comput. Ind. Eng..

[35] Liu C.Y., Xia T.S., Tao Y.U., School B., University S.N. (2019). Research on Evolutionary Game of Government Regulation and Production of Manufacturer in the Angle of Low Carbon. Chin. J. Manag. Sci..

[36] Zhang L., Song Y., Zhang M., Wu W. (2022). Evolutionary game analysis of strategic interaction of environmental regulation among local governments. Environ. Dev..

[37] Ren J., Ping H.E., Gong B.G., Management S.O. (2016). Game Analysis between Government and Enterprises and the Strategy of Government Subsidies under Low-Carbon Economy. Oper. Res. Manag. Sci..

[38] Fan S., Wang Q., Li C., Management S.O. (2017). A Study on Emission Reduction Game of Three Level Low Carbon Supply Chain under Government Subsidies. J. Ind. Technol. Econ..

[39] Tian Y., Govindan K., Zhu Q. (2014). A system dynamics model based on evolutionary game theory for green supply chain management diffusion among Chinese manufacturers. J. Clean. Prod..

[40] Liu Z., Qian Q., Hu B., Shang W.-L., Li L., Zhao Y., Zhao Z., Han C. (2022). Government regulation to promote coordinated emission reduction among enterprises in the green supply chain based on evolutionary game analysis. Resour. Conserv. Recycl..

[41] Yang L., Xu M., Yang Y., Fan J., Zhang X. (2019). Comparison of subsidy schemes for carbon capture utilization and storage (CCUS) investment based on real option approach: Evidence from China. Appl. Energy.

[42] Fan J.L., Xu M., Wei S., Shen S., Diao Y., Zhang X. (2020). Carbon reduction potential of China’s coal-fired power plants based on a CCUS source-sink matching model. Resour. Conserv. Recycl..

[43] Li X., Yao X. (2020). Can energy supply-side and demand-side policies for energy saving and emission reduction be synergistic?--- A simulated study on China’s coal capacity cut and carbon tax. Energy Policy.

[44] Taylor P.D., Jonker L.B. (1978). Evolutionarily Stable Strategies and Game Dynamics. Math. Biosci..

[45] Weinstein M.I. (1986). Lyapunov Stability of Ground States of Nonlinear Dispersive Evolution Equations.

[46] Rubin E.S., Davison J.E., Herzog H.J. (2015). The Cost of CO 2 Capture and Storage. Int. J. Greenh. Gas Control.

[47] Yuan X., Chen L., Sheng X., Liu M., Xu Y., Tang Y., Wang Q., Ma Q., Zuo J. (2021). Life Cycle Cost of Electricity Production: A Comparative Study of Coal-Fired, Biomass, and Wind Power in China. Energies.

[48] Ystad P.A.M., Lakew A.A., Bolland O. (2013). Integration of Low-Temperature Transcritical CO_2_ Rankine Cycle in Natural Gas-Fired Combined Cycle (NGCC) with Post-Combustion CO_2_ Capture. Int. J. Greenh. Gas Control.

[49] Luo X., Wang M. (2016). Optimal Operation of MEA-Based Post-Combustion Carbon Capture for Natural Gas Combined Cycle Power Plants under Different Market Conditions. Int. J. Greenh. Gas Control.

[50] Fan J.L., Shen S., Xu M., Yang Y., Zhang X. (2020). Cost-Benefit Comparison of Carbon Capture, Utilization, and Storage Retrofitted to Different Thermal Power Plants in China Based on Real Options Approach. Adv. Clim. Chang. Res..

[51] Jiao J.L., Chen J., Li L., Li F. (2017). A Study of local governments’ and enterprises’ actions in the carbon emission mechanism of subsidy or punishment based on the evolutionary game. China J. Manag. Sci..

[52] Zheng S., Yu L. (2022). The government’s subsidy strategy of carbon-sink fishery based on evolutionary game. Energy.

[53] Rubin E.S., Azevedo I.M.L., Jaramillo P., Yeh S. (2015). A review of learning rates for electricity supply technologies. Energy Policy.

[54] Report S., Utilisation C.C. (2020). Energy Technology Perspectives.

[55] Guo H., Davidson M.R., Chen Q., Zhang D., Jiang N., Xia Q., Kang C., Zhang X. (2020). Power market reform in China: Motivations, progress, and recommendations. Energy Policy.

[56] Helgesen P.I., Tomasgard A. (2018). An equilibrium market power model for power markets and tradable green certificates, including Kirchhoff’ s Laws and Nash-Cournot competition. Energy Econ..

[57] Wang W., Zhou C., Li X. (2019). Carbon reduction in a supply chain via dynamic carbon emission quotas. J. Clean. Prod..

